# Genetic Diversity in C-terminal of SERA5 Gene in the Blood Stage of Human Isolates of *Plasmodium vivax* in Sistan and Baluchistan, Iran

**Published:** 2018

**Authors:** Ahmad ABOLGHAZI, Aliehsan HEIDARI, Vahideh MOIN-VAZIRI, Ali HAGHIGHI, Seyyed Javad SEYYED TABAEI, Hossein KESHAVARZ, Saeedeh SHOJAEE

**Affiliations:** 1. Dept. of Parasitology and Mycology, School of Medicine, Shahid Beheshti University of Medical Sciences, Tehran, Iran; 2. Dept. of Medical Parasitology, School of Medicine, Alborz University of Medical Sciences, Karaj, Iran; 3. Dept. of Medical Parasitology and Mycology, School of Public Health, Tehran University of Medical Sciences, Tehran, Iran; 4. Center for Research of Endemic Parasites of Iran (CREPI), Tehran University of Medical Sciences, Tehran, Iran

**Keywords:** *Plasmodium vivax*, PvSERA5, Genetic diversity, Iran

## Abstract

**Background:**

*Vivax* malaria is more prevalent in the malarious areas of Iran, which makes vaccine research a high priority. Serine Repeat Antigens (SERA) have essential role in the parasite life cycle and high expression profiles of PvSERA5 make it suitable vaccine candidates. This study aimed to evaluate the genetic diversity of C-terminal region of PvSERA5 in Iranian isolates of *Plasmodium vivax* in Sistan and Baluchistan.

**Methods:**

Totally, 49 blood samples were taken from symptomatic malaria patients in Sistan and Baluchistan Province in 2016. Mono-infection to *P. vivax* was confirmed by 18srRNA-Nested-PCR. Genomic DNA was extracted and C-terminal region of PvSERA5 was amplified by specific primers. PCR-products have been sequenced and analysis was done by using bioinformatics software, mainly DnaSP & MEGA5.

**Results:**

Genetic diversity was calculated 14.8% in C-terminal region of PvSERA5 in Iranian isolates, 19 different sequences and 4 haplotypes existed. The amount of Tajima’s D (0.3805) and ratio of non-synonymous to synonymous mutation (1.82) showed that C-terminal region of PvSERA5 is under positive natural selection; also intragenic recombination could interfere.

**Conclusion:**

Results could be helpful in any research, regarding this antigen as vaccine candidate in Iran or worldwide.

## Introduction

Despite global efforts against malaria, it is still one of the health challenges worldwide, based on WHO report, about 212 million cases of disease occurred globally (range 148–304 million), with 429 000 deaths (range 235000–639000) (1). Among five *Plasmodium* species, which cause malaria in humans, *P. falciparum* and *P. vivax*, pose the greatest threat. *P. falciparum* is the most prevalent malaria parasite on the African continent. It is responsible for most malaria-related deaths globally, but *P. vivax* is the dominant malaria parasite in most countries outside of sub-Saharan Africa, so it is the most geographically distributed causative agent of the disease (1, 2).

Iran is classified as being in the malaria elimination phase among 91 countries with ongoing malaria transmission (1). Most problematic areas in Iran are located in the south and southeast of the country, bordered by Pakistan and Afghanistan. Two causative agents of disease exist sympatrically in these areas; *P. falciparum*, fatal form of disease and *P. vivax*, predominate form of disease (3–5). Totally, 1378 confirmed cases were reported countrywide (6).

Although vivax malaria is also prevalent in the world, major attention in vaccine research goes to the *P. falciparum* due to its importance. Moreover, management of *vivax* malaria has been encountered with some difficulties due to the drug resistance, clinical patterns and its relapse, genetic diversity, isoenzyme markers and microsatellite poses (7). One of the crucial problems in vaccine development against malaria is antigenic diversity, seen more in the antigen-encoding genes compared to other protein-coding genes (7). Understanding the genetic variation is essential in any vaccine efficacy trial (8), among the different asexual blood stage antigens of *P. vivax*, Merozoite Surface Protein-1 (MSP-1), Duffy Binding Proteins (DBP) and Apical Membrane Antigen (AMA)-1 received more attention (9–11).

Among the different vaccine candidates, Serine Repeat Antigens (SERA) which highly expressed through the late trophozoite to schizont, in the blood stage of the parasite, seems promising, as they have essential role in the parasite life cycle. In comparison to *P. falciparum* SERA (PfSERA), limited data are available about *P. vivax* (PvSERA). In *P. vivax,* they belong to a multigene family, with 12 homologues (12–14). The former research had some contradiction results, only PvSERA5 was transcribed (15), following studies reported that PvSERA4 was the most transcribed member, then PvSEAR2, 5, 10 and 11 (12). High expression profiles of PvSERA5 make it suitable vaccine candidates. Most of divergence exists in a 200 amino acid stretch in the C-terminal region of PvSERA genes (15). Similarly, genetic diversity of PvSERA localized in the C-terminal region of the proteins (12). Genetic diversity and the selection pattern studies seem all-important in each malarious areas.

There is no information on genetic diversity of PvSERA5 in Iran, therefore, this study aimed to evaluate the genetic diversity of C-terminal of PvSERA5 in Iranian isolates of *P. vivax* in Sistan and Baluchistan.

## Materials and Methods

### Blood collection from field

**Fig. 1:**
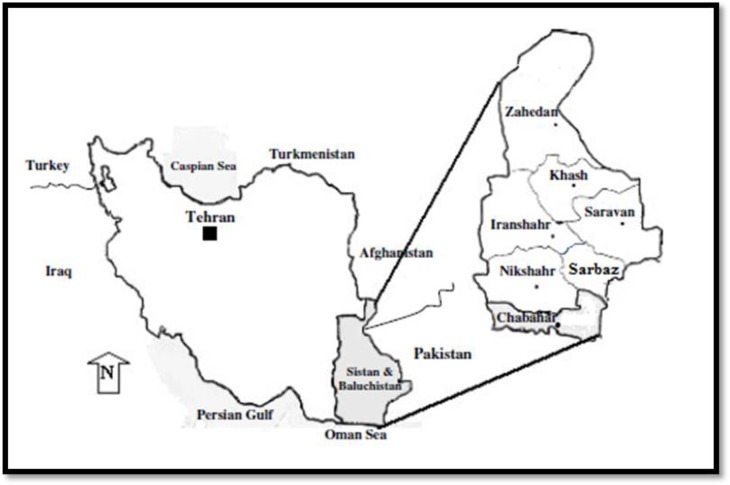
Map showing the geographical situation of Chabahar County located in the southern part of Sistan & Baluchistan province, Iran

Blood samples were taken from symptomatic malaria patients referred to malaria centers in Sistan and Baluchistan Province (Fig. 1). Although Giemsa stained blood slides were screened carefully by microscope at 1000× magnification to discharge mixed infection, Nested PCR was also applied using the *Plasmodium* 18 subunit ribosomal ribonucleic genes to detect vivax mono-infection.

Only, the confirmed vivax positive cases were used and totally 49 patients participated in this study. Whole blood samples were poured into EDTA coating tubes and stored at −20 °C. An informed consent was taken from each patient.

### DNA Extraction

Genomic DNA was extracted from 200 μL blood samples by using QIAquick PCR purification kit (Qiagen, Germany), according to the manufacturer’s instruction.

### SERA gene amplification

The forward (5′GCGCGGGAAGAAGGTGCAAAG3′) and reverse (5′GCGCCCGTCACACTCTTCCTAC3′) primers were used to amplify C-terminal region of PvSERA5 (13). The PCR premix was from Bioneer Kit, amplification reactions were set for a final volume 25μ, containing 5 μl premix, 2 μl forward and reverse primers (10 pmol), 2 μl DNA templates and 16 μl doubled distilled water. The cycling parameter included an initial step of denaturation 95 °C for 3 min, followed by 35 cycles (denaturation 94 °C for 1 min, the annealing 59 °C for 1 min 30 sec, then extension 72 °C for 2 min 30 sec), with a final extension 72 °C for 10 min.

After electrophoresis on gel agarose 1.5%, PCR products were purified and sequenced using forward and reverse primers.

### Bioinformatics analysis

The sequences were checked manually for ambiguities by Chromas software (ver. 2.33). To find similarities, DNA sequences were compared with sequences in GenBank database using Basic Local Alignment Search Tool (BLAST) analysis (https://blast.ncbi.nlm.nih.gov/Blast.cgi). Nucleotide sequences data were submitted to the GenBank under accession numbers KY363298-KY363315 and KY290841. ClustalW program was used to align the nucleotide sequences to each other (http://www.genome.jp/tools/clustalw/). DNaSP version 5.1 and MEGA5 software packages were used to analyse genetic diversity of the SERA5 gene.

### Ethical Considerations

Ethics clearance was obtained from the Research Ethical Community of Shahid Beheshti University of Medical Sciences (Approval Number: IR.SBMU.MSP.REC.1395.122).

## Results

**Fig. 2:**
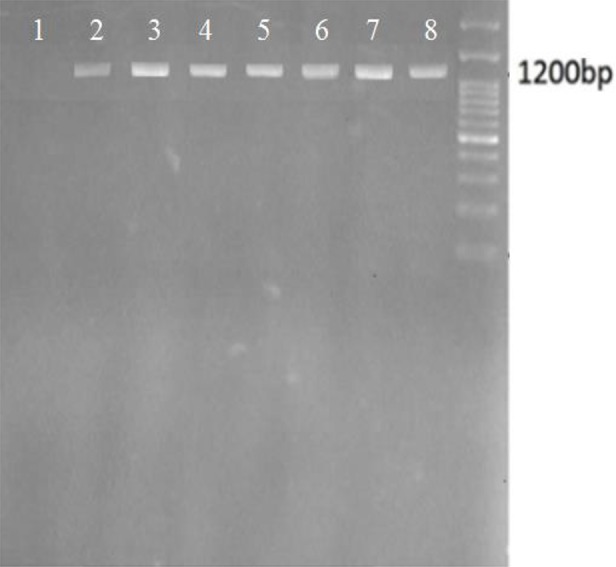
Electrophoresis of C-terminal region of SERA5 of *Plasmodium vivax* isolates of malaria patients in Sistan & Baluchistan province, Iran, 2014– 2015. Lane 1: Negative control, Lane 2–7: Samples of current study, Lane 8: Last lane: 100 bp ladder marker (CinnaGen Co.)

A single product of about 1200 bp has been amplified in all samples (Fig. 2). There was no variation size in PCR products. Among 49 amplified samples, 19 different sequences existed. The analysis was carried out on the C-terminal region of PvSERA5 of these 19 sequences which was about 351 bp (corresponding to 2134-2565 bp region of Sal-1 SERA5 gene encoding for amino acid from 714 to 838).

Using BLAST, 89%–100% homology was observed between the sequence of our samples and registered sequences of *P. vivax* from Strain Salvador1(Central America) and India isolate, which respectively are accessible by following accession numbers in the GenBank, XM001612955, and JQ956488.

### Analysis at nucleotide and amino acid level

At nucleotide level, there were 299 (85.2%) monomorphic (invariable) sites, and 52 (14.8%) sites were polymorphic (variable). Among 52 polymorphic sites, one was singleton and 51 found to be parsimony informative (one site tri-morphic and others dimorphic). The 53 point mutations appeared that 34 were nonsynonymous. These variations at nucleotide level caused changes at 34 resulting amino acids out of 113 ones. Pi which represents the nucleotide diversity was 0.04723 (0.040 ± 0.00588 SD).

### Haplotypes analysis

Among 19 isolates, four different haplotypes with different frequency were observed. The frequency of haplotype 1, 2, 3 and 4 were 68.4%, 10.5%, 5.3% and 15.8% respectively.

Using the BLAST, search against all PvSERA5 deposited in GenBank, six isolates are globally novel PvSERA5 which represented by following accession numbers KY363300, KY363303, KY363306, KY363310, KY363312, and KY363314. The haplotype diversity (Hd) was found to be 0.5205 (0.0123 ± 0.01510 SD), which indicates the low amount of diversity among PvSERA5 of Iranian field isolates of *P. vivax* collected from Chabahar County, Iran.

### Phylogenetic Analysis

**Fig. 3:**
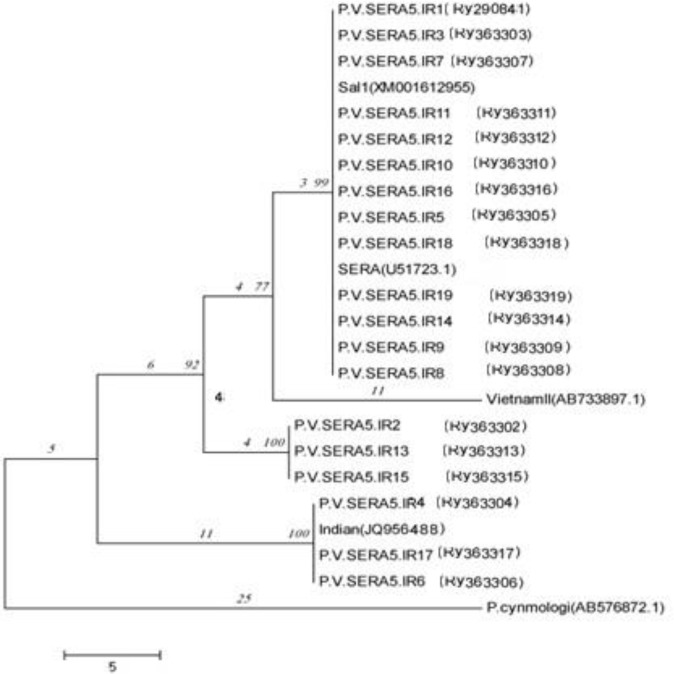
Phylogenetic tree of C-terminal region of PvSERA5 sequences. Neighbor-Joining tree was used for tree construction of isolates from Iran with Sal I isolate and few samples from India and Vietnam. The partial sequence of *Plasmodium cynomolgi* was applied as an outgroup species

The phylogenetic relationship of Iranian isolates of PvSERA5 was checked with Salvador-1 strain and isolates from other countries accessible from GenBank (Salvador-1 strain: XM001612955, Central America isolate: U51723.1, Indian isolates: JQ956488, Vietnamese isolate: AB733897.1) by utilizing the Neighbor method Joining (NJ) with 1000 replication as bootstrap (Fig. 3).

### Neutrality test and recombination analysis

The achieved Tajima’s D test from DnaSP software (ver. 5) was 0.3805, the positivity showed that C-terminal region of PvSERA5 is under positive natural selection. Moreover, the ratio of non-synonymous to synonymous mutation was calculated as 1.82, which showed that most of the mutation was non-synonymous and would influence on amino acids, so, predict positive selection in C region of PvSERA5. The finding of Tajima’s test another neutrality test was 0.3805 that illustrated positive natural selection events in this section of SERA5 gene.

**Fig. 4:**
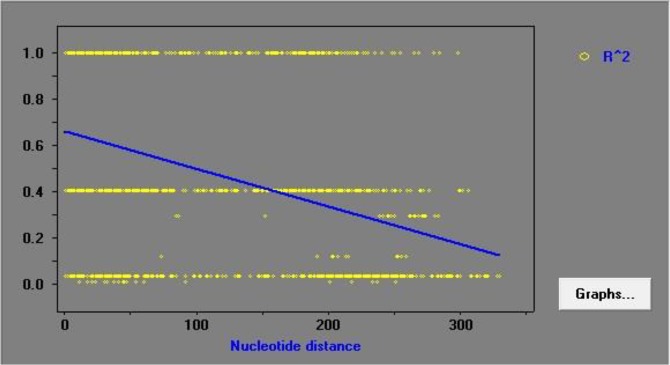
The linkage disequilibrium (LD) index obtained from analysis of C-terminal sequence region of Iranian isolates of PvSERA5

Fig. 4 showed the effect of recombination on the genetic diversity of C-terminal region of PvSERA5 in current study isolates. From declining trend of LD (Linkage Disequilibrium) level (R^2^ indices) with increasing nucleotide distance between pairs of nucleotide sites illustrated that intragenic recombination could also interfere on the genetic diversity of C-terminal region of PvSERA5.

**Fig. 5:**
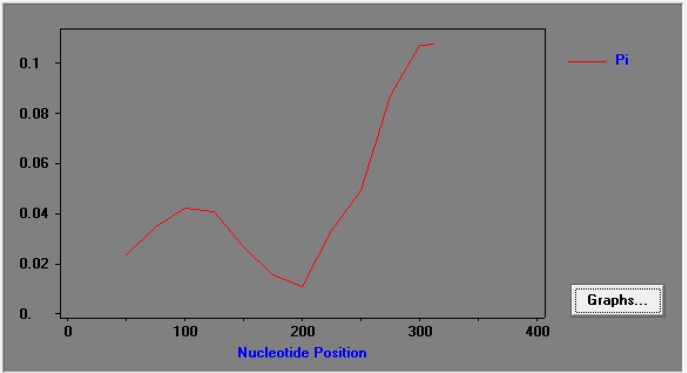
The sliding window plot of nucleotide diversity, Pi (p) of C-terminal sequence region of Iranian isolates of PvSERA5

The sliding window method using a window length of 100 bp and step size of 25bp showed π diversity ranging from 0.02575 to 0.06867 Maximum variation occurred between nucleotide sites 249 (Fig. 5). In the C terminal SERA5 gene, the average number of pairwise nucleotide differences (K) was 0.42880. Overall, 34 polymorphic amino acid sites occurred at 113 positions.

## Discussion

*Plasmodium vivax* causes significant morbidity and mortality and it has the widest geographical distribution of human malaria and accounts for about half of malaria cases outside sub-Saharan Africa (16). The biology of *P. vivax* presents several challenges in its control compared to *P. falciparum*; a) it is able to form hypnozoites which emerge months to years later to cause blood stage infections, b) its gametocytes appear earlier than clinical symptoms and finally c) it has shorter developmental cycle in the Anophelinae vector. These facts make several challenges in its elimination using standard control tools (16, 17). The availability of an effective vaccine that provides protection and prevents transmission would be a valuable tool to control of malaria (18, 19). Several vaccine candidates introduce up to now, here we present the genetic diversity of PvSERA5 as a promising candidate among Iranian isolates of *P. vivax* collected from Chabahar County, Sistan and Baluchistan Province, Iran.

One of the dominant transcribed members of SERA gene in *P. vivax* is SERA5 which highly expressed through the late trophozoite to schizont, in the blood stage. The main goal of present study, which is the first of its kind in Iran, was studying the genetic polymorphism of the most variable part of PvSERA5, C-terminal region.

Genetic diversity analysis of C-terminal region of PvSERA5 in the field Iranian isolates indicates some nucleotide variation alongside with different amount of deletion and insertion. Nucleotide diversity (Pi) was calculated 0.04723 in current study, most of the polymorphic sites are dimorphic, moreover, one tri-morphic and one singleton substitution were observed. Variation in polymorphism sites showed that the parasite partakes from nucleotide substitutions to create allelic diversity (20, 21), which is essential point in antigens considered as vaccine candidate. Diversity in the C-terminal region of PvSERA5 is also in accordance to the previous researchers finding (22, 23). In the most interrelated work to our study, the given amount for nucleotide diversity among Indian field isolates was 0.17229±0.02257 (13), which is much higher than what we obtained; it could be due to the higher malaria prevalence and transmission in India. An outcome of current study was introducing 6 novel isolates among 49 Iranian deposited isolates in the Genbank. Overall, 15 novel haplotypes among 18 sequences in Indian isolates in 2013 (13), the difference could be due to the mentioned above reason and decreasing trend of indigenous malaria transmission in Iran. The role of positive natural selection outweighs the recombination in the genetic diversity of PvSERA5 (13).

The same deduction from our analysis; the positive value of Tajima’ D test which is consequent of higher non-synonymous substitutions than synonymous ones (dN>dS), is the sign of positive natural selection (13, 24).

Intragenic recombination is another cause of genetic diversity, influenced by sexual out-crossing of the parasite which happened in the midgut of *Anopheles* spp. (25), the hypothesis could be supported by declining trend of LD index with increasing nucleotide distance in current analysis.

Neighbor-joining tree constructed based on C-terminal sequence of PvSERA divided the 19 Iranian isolates into four haplotype at three different clades. The second and the third clades were separated from each other by having 21 and 19 shared polymorphic sites respectively and from the first branch by having 11 shared polymorphic sites. A Vietnamese isolate (AB733897.1) was set as sister group of the first clade and Salvador I strain (XM001612955) and one isolates from Central America (U51723.1) were located in the first clade and an Indian isolate (JQ956488) was situated in the third clade. Considering the malaria status in Iran 1) having encountered hypo-endemic malaria and 2) being in the elimination phase of control, provoke the expectation of having low amount of genetic diversity and haplotypes. The problematic malarious areas in Iran highly was affected by the disease in the neighboring countries, Afghanistan and Pakistan and undoubtedly would influence the genetic construction of *Plasmodium* spp. (26). Therefore, the extending knowledge on the genetic variation of any malaria vaccine candidate in the adjacent countries seems crucial for having an effective regional vaccine.

## Conclusion

This is the first report on genetic characterization of PvSERA5 in field isolates from Sistan & Baluchistan, Iran. Results could be helpful in any research, regarding this antigen as vaccine candidate in Iran or worldwide.
